# ITGA1 Promotes Glioma Cell Proliferation and Affects Immune Cell Infiltration in Low-Grade Glioma

**DOI:** 10.1155/2024/6147483

**Published:** 2024-10-29

**Authors:** Yanhong Ren, Jianchang Xu, Zhengkui Zhang, Rutong Yu

**Affiliations:** ^1^Department of Neurosurgery, The Affiliated Hospital of Xuzhou Medical University, West Huai-Hai Road 99, Xuzhou, Jiangsu, China; ^2^Department of Neurosurgery, The Affiliated Huai'an Hospital of Xuzhou Medical University, South Huai-Hai Road 62, Huai'an, Jiangsu, China

**Keywords:** immune infiltration, ITGA1, LGG, prognostic, survival analysis

## Abstract

**Background:** Low-grade glioma (LGG) is a commonly occurring type of central nervous system cancer. Integrin α1 (ITGA1), a family member of integrins, is implied in the malignant development of cancers, but the fundamental role of ITGA1 has not been illustrated yet in glioma. This study aimed to evaluate the prognostic value of ITGA1.

**Methods:** Correlations between ITGA1 and relevant clinical features were analyzed in the LGG datasets based on Chinese Glioma Genome Atlas (CGGA) and Tumor Genome Atlas (TCGA). Glioma clinical samples and glioma cell lines were identified at the level of mRNA and protein level by Western blot. Cox regression were developed to assess the involvement of ITGA1 expression in predicting survival in LGG patients. Application of GSEA enrichment analysis to reveal ITGA1-mediated biological functions in LGG. Using TIMER 2.0 to analyze correlations between immune cell infiltration. In addition, ITGA1 high expression was analyzed for correlation with immune checkpoint-related genes and cumulative survival time.

**Results:** ITGA1 was significantly more expressed in LGG than in normal samples. Cox regression indicated that ITGA1 was a risk factor independently for prognosis in LGG patients. GSEA enrichment analysis indicated that ITGA1 was engaged in several immunomodulatory processes. ITGA1 expression was shown to be highly correlated with the immune score, stromal score, and estimate score in LGG. ITGA1 was significantly related to the immune checkpoint-associated gene expression. In vivo experiments showed that overexpression of ITGA1 promoted glioma cell invasion.

**Conclusion:** High ITGA1 expression is correlated with immune infiltration of the low-grade tumor, leading to poor prognoses in LGG patients.

## 1. Introduction

The glioma is a malignant tumor of primary origin in the central nervous system, which accounts for over 80% of intracranial tumors [1]. Glioma is classified as low-grade glioma (LGG, World Health Organization (WHO) grade 2) and high-grade glioma (HGG, WHO grade 3–4) according to the WHO histopathological classification criteria [2]. LGG includes astrocytoma and oligodendroglioma, which have a tendency to progress into HGG [3]. Despite the current comprehensive treatment strategy of surgical excision with radiation therapy and chemotherapy, the prognosis for LGG patients is still poor and tumor recurrence is almost inevitable. Thus, identifying effective prognostic indicators and emerging therapeutic targets for gliomas is of great significance.

The tumor microenvironment (TME) plays a critical role in tumorigenesis [4]. At the early stage, immune cells inhibit tumor progression and expansion by activating immune responses [5]. With the tumor progressing continuously, the TME changes in composition, and some tumor cells can escape from the removal by the immune system, leading to immune tolerance and tumor progression [6, 7]. As an emerging therapeutic approach, immunotherapy has been applied to treat a range of cancers. Targeting signaling molecules such as the immune checkpoint PD-1 and CTLA4 has proved to be the most effective approach in gastric cancers [8, 9]. However, there is a lack of effective immunotherapeutic targets for the treatment of glioma.

Integrin *α*1 (ITGA1), a family member of integrins, is involved in regulating intercellular signaling transduction by binding with the extracellular matrix (ECM) and thus plays a crucial role in the proliferation of tumor cells, and migration [10]. Aberrant activation of ITGA1 is inextricably related to the development of various cancers. It has been shown that ITGA1 induces epithelial–mesenchymal transition (EMT) and enhances the tumorigenic potency in pancreatic carcinoma cells [11]. ITGA1 promotes lung cancer cell proliferation and metastasis [12]. Known down ITGA1 reduces tumor cell invasion and adhesion in breast cancer [13]. Similar findings have also been reported in metastatic melanoma [14] and hepatocellular carcinoma (HCC) [15]. However, it is less well known about the role of ITGA1 in gliomas and the relationship of ITGA1 with tumor immunity.

In this research, we downloaded the LGG datasets from the Tumor Genome Atlas (TCGA) and the Chinese Glioma Genome Atlas (CGGA). The connection between ITGA1 expression levels and LGG clinical features was analyzed to determine its potential function and prognostic value. In addition, we assessed the relationship in which ITGA1 was associated with immune checkpoint-associated gene.

## 2. Material and Methods

### 2.1. Data Collection and Processing

We first downloaded from the TCGA (http://www.tcga.org) RNA-seq and associated clinical information for LGG samples. Log2 conversion of RNA-seq data by R package.

Expression profiles of genes and corresponding clinical information for the mRNA-seq_325 datasets were obtained from the CGGA (http://www.cgga.org.cn/). Cases with insufficient or missing data on age and overall time of survival were removed.

### 2.2. Collection of Clinical Glioma Samples

Clinical tissue samples derived from the Affiliated Hospital of Xuzhou Medical University were collected. The tissue specimens were gained by surgical excision and were not radiotherapy and chemotherapy-treated (Grade 2, *N* = 9; Grade 3, *N* = 11; Grade 4, *N* = 9), nontumor brain tissue specimens derived from patients who underwent traumatic brain injury decompression surgery (nontumor, *N* = 8). The clinical pathology of these subjects is shown in Table S1 and it was used in our previously published paper [16]. All patients signed an informed consent and were approved by the Ethics Committee of the Affiliated Hospital of Xuzhou Medical University (Approval No. XYFY2022-KL296). Glioma specimens had confirmed pathological diagnoses and were classified in accordance with WHO criteria.

### 2.3. ITGA1 Expression Correlates With Prognosis

Differential expression of ITGA1 in several tumor types was analyzed in the TCGA database. We applied Gliovis online database (http://gliovis.bioinfo.cnio.es/) to the TCGA and CGGA LGG datasets and analyzed the association between ITGA1 and other revalent clinical features such as WHO grade, IDH-1, and 1p/19q based on the TCGA and CGGA LGG datasets. We applied glioma tissue specimens and glioma cell lines to validate ITGA1 mRNA levels by quantitative PCR (qPCR). We used glioma tissue specimens to analyze ITGA1 protein levels by the Western blot and applied the HPA database (https://www.proteinatlas.org/) to access the difference between normal tissue and glioma tissue. In addition, we developed Cox regression analysis models that are based on the CGGA LGG dataset to assess whether ITGA1 was one of the independent factors that can affect the prognosis of LGG patients.

### 2.4. Cell Culture

LN229, U87, U251, and HA1800 cells were obtained from the ATCC cell bank (Shanghai, China). All cell lines were incubated in Dulbecco's modified Eagle's medium (DMEM, Biochannel, Nanjing, China) which contains 10% fetal bovine serum (FBS, Biochannel). All cell lines were incubated that contained 5% CO_2_ at a temperature of 37°C.

### 2.5. Cell Transfection

Lentiviral vector were constructed. The targeting sequences are as follows: NC (5′-UUCUCCGAACGUGUCACGUTT-3′), sh-1(5′-CCUAUAGAUGUGGACAUUUTT-3′), sh-2(5′-GCUCUAGUCACCAUUGUUATT-3′), and sh-3(5′-GCACUAAGCACUCCUUCUATT-3′). Cell transfection was performed with PolyJet, according to the manufacturer's operating instructions. Lentivirus was produced by 293T cells.

### 2.6. RNA Extraction and Real-Time qPCR (RT-qPCR)

The glioma samples information is shown in Table S1. RNA extraction and reverse transcription of cDNA were performed by Vazyme (Vazyme Biotech, Nanjing, China) following the instructions of the kit. RT-qPCR was run by Universal Blue SYBR Green qPCR Master Mix (Servio, Wuhan, China). The primer sequences were designed as follows: ITGA1 (forward: 5′-GGTGCTTATTGGTTCTCCGTTAG-3′ and reverse: 5′-CTCCTTTACTTCTGTGACATTGGG-3′) and GAPDH (forward: 5′-AACGAACACAAGTTACCTATC-3′ and reverse: 5′-ACATGAGGGAAACCGAGGG-3′). Expression levels of ITGA1 were normalized to GAPDH and were determined by the 2^−ΔΔ*Ct*^ method.

### 2.7. Western Blot Analysis

The glioma samples information is shown in Table S1. The protein was extracted from glioma tissue samples. Western blotting was executed according to the description in our published paper [17]. ITGA1 antibody (Proteintech, Wuhan, China) was diluted at 1:1000, and *β*-actin (1:4000, Proteintech, Wuhan, China) was used as a loading control. The fragment density was measured using ImageJ software.

### 2.8. Gene Function Analysis of Enrichment

GSEA software 20 (V version 4.1.0) was used to reveal the biological process in which ITGA1 may be involved [18]. LGG sample in the TCGA dataset was grouped into ITGA1 low expression and high expression groups with parameters set to a *p* value of less as compared to 0.05, |enrichment scoring (ES)| greater as compared to 0.5, gene size of greater as compared to 50, and false discoveries rate (FDR) of less as compared to 0.05 for KEGG pathway enrichment analysis.

### 2.9. Immune Cell Infiltration Analysis

We employed the ESTIMATE methods to assess the relative abundance of infiltrating immune cells concerning ITGA1 expression. The correlation between ITGA1, the infiltrating immune cell abundance, and the survival time of LGG patients was also analyzed in the TCGA LGG dataset. In addition, a correlation analysis between ITGA1 and immune checkpoint-related genes was performed.

### 2.10. Clone Formation Experiment

Glioma cells (1000 cells/well) were spread in six-well plates, after incubation for 14 days, stained with 0.1% crystal violet, and photographed.

### 2.11. Transwell Invasion Assay

Cultured cells were resuspended in the upper chamber and cultured into a transwell treated with matrix gel (Corning, New York, USA) with a pore size of 8 μm containing serum-free medium. DMEM medium containing 10% serum was injected into the bottom chamber, after incubated for 36 h in culture incubator.

### 2.12. EdU Cell Proliferation Assay

Glioma cells were grown in 96-well plates (5000 cells/well) cultured for 24 h in cell culture incubator. The EdU proliferation was conducted in accordance with the kit instructions.

### 2.13. Statistical Analysis

R software (V version 4.0.3) and SPSS software (V version 26.0.0) were used to generate statistical analyses and charts. Cox regression models were established for one-way and multi-way analyses. Variations of multiple groups were analyzed by one-way ANOVA and Dunnett's or Tukey's post hoc test by GraphPadPrism 8. Variation in survival was estimated by the Kaplan–Meier method. *p*  < 0.05 was regarded as the statistically meaningful variance.

## 3. Results

### 3.1. ITGA1 Expression Correlates With the WHO Grade, IDH1, and 1p19q Status in LGG

**Figure 1:**
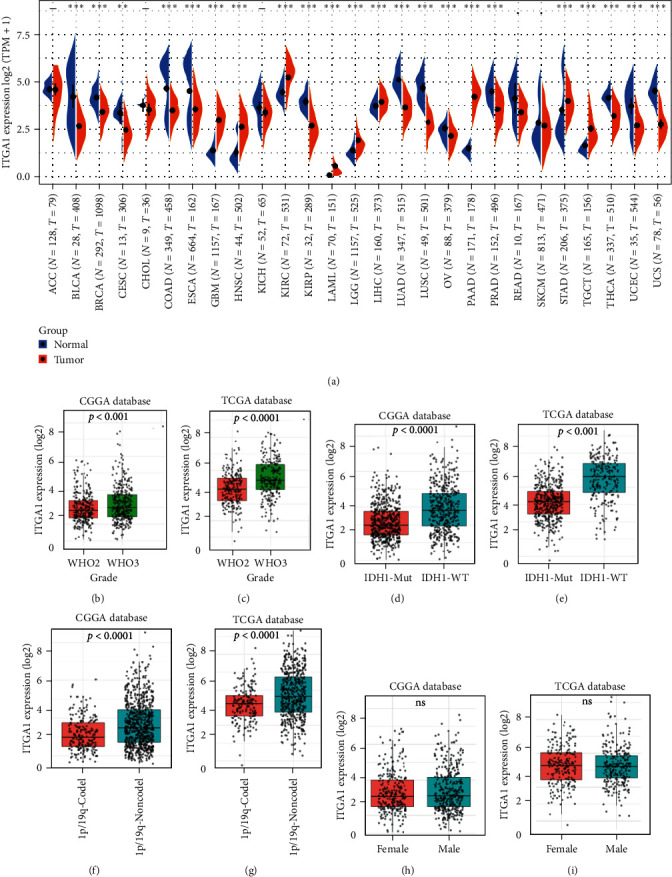
Expression analysis of ITGA1 mRNA in public glioma datasets. (A) Pan-cancer analysis of ITGA1 mRNA expression in the TCGA database. (B, C) Differential expression analysis of ITGA1 between WHO grade 2 and 3 gliomas. (D, E) Differential expression analysis of ITGA1 between IDH-1-wildtype (WT) and IDH1-mutant (Mut). (F, G) Differential expression analysis of ITGA1 between 1p/19q-Codel and 1p/19q-Noncodel. (H, I) Differential expression analysis of ITGA1 between males and females. *⁣*^*∗∗*^*p* < 0.01, *⁣*^*∗∗∗*^*p*  < 0.001; ns, not significant.

A pan-cancer analysis showed that ITGA1 expression was higher in GBM, PAAD, KIRC, LGG, LIHC, OV, HNSC, while it was lower in BRCA, PRAD, BLCA, ESCA, KIRP, LUAD, CESC, LUSC, COAD, STAD, THCA, and UCS in the TCGA dataset when compared to the corresponding normal tissue (Figure 1A). We next analyzed the correlation of ITGA1 expression with WHO grades, IDH1, and 1p19q status, and sex based on the LGG dataset obtained from TCGA and CGGA. ITGA1 was obviously higher in grade 3 than in grade 2 glioma (Figure 1B,C), and it was upregulated significantly in IDH1 wild-type and 1p19q non-codeficient LGG as compared to IDH mutant and 1p19q codeficient LGG. respectively (Figure 1D–G). ITGA1 expression did not obviously difference between the gender (Figure 1H,I).

**Figure 2:**
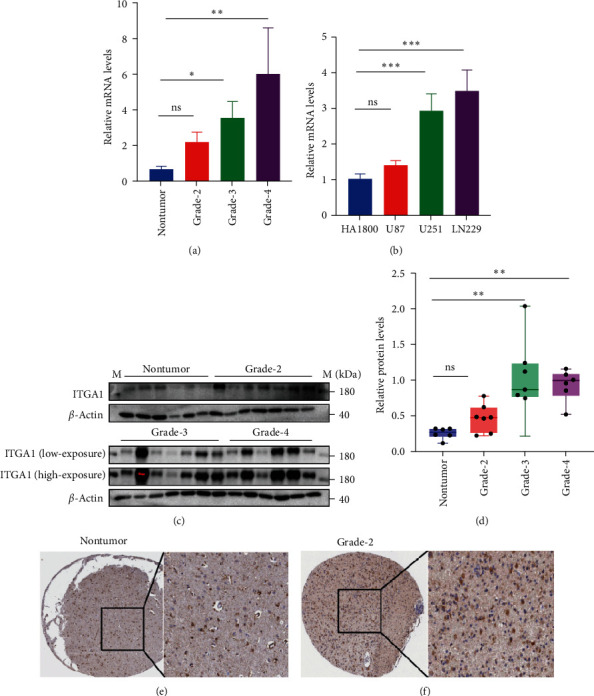
Expression analysis of ITGA1 in our glioma samples. qPCR assay showed the changes of ITGA1 mRNA levels in different grades of glioma tissues (A) and in different glioma cell lines (B). (C, D) Western blot analysis showed the changes of ITGA1 protein levels in different grades of glioma tissues. (E, F) Immunohistochemistry exhibited the ITGA1 immunoreactivity in nontumor brain tissues and WHO grade 2 glioma tissues. Bar: 100 μm. Data was obtained from the HPA database. M, protein marker; *⁣*^*∗*^*p*  < 0.05, *⁣*^*∗∗*^*p*  < 0.01; *⁣*^*∗∗∗*^*p*  < 0.001; ns, not significant.

To confirm the increased expression of ITGA1 in glioma tissues, we performed qPCR and Western blot to measure the mRNA and protein levels of ITGA1 in our glioma cohort. qPCR assay showed that ITGA1 mRNA level was increased with the increase of glioma grades, and it was relatively higher in glioma cell lines than in HA1800, a human astrocyte cell line (Figure 2A,B). Western blot analysis showed that ITGA1 was upregulated in glioma tissues compared to nontumor brain (Figure 2C,D). Immunohistochemical data available from the HPA showed that ITGA1 immunoreactivity was obviously increased in LGGs compared to nontumor brain tissues (Figure 2E,F; Figure S1). Therefore, the results indicate that ITGA1 is positively related to the malignant degree of LGG.

### 3.2. ITGA1 Is Related to Inferior Prognosis in LGG Patients

**Figure 3:**
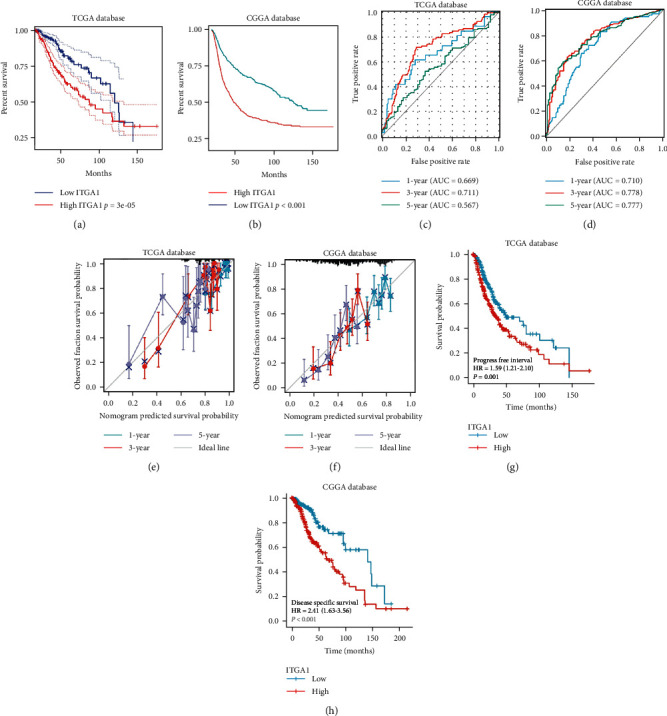
Prognostic values of ITGA1 in LGG. (A, B) Kaplan–Meier analysis of the association of ITGA1 expression with the survival of LGG patients. (C, D) ROC curve analysis of the reliability of ITGA1 in predicting survival in LGG patients. (E, F) Calibration analysis of the accuracy of ITGA1 in predicting LGG patients' survival. (G, H) Correlation analyses of ITGA1 expression with progression-free interval and disease-specific survival in LGG patients from the TCGA database.

To investigate whether high expression of ITGA1 could predict the prognosis in LGG, we collected gene-expressed profiles and relevant clinical information from the TCGA and CGGA databases. Depending on the level of median ITGA1 expression, patients were grouped into high and lower. Cox regression analysis based on the CGGA dataset showed that chemo-status, 1p19q-codeletion, and high ITGA1 expression are independent prognostic risk factors in LGG (Table S2). Kaplan–Meier survival analysis indicated that LGG patients in the low ITGA1 expression group had longer overall survival than in the high expression group (*p*  < 0.0001, Figure 3A,B). Validating the predictive value of ITGA1 expression on the survival of LGG patients, we analyzed a receiver operating characteristic (ROC) curve analysis. In the TCGA and CGGA database, the AUC for 1, 3, and 5-year follow-ups was >0.710 (Figure 3C,D). Calibration analysis further reveals that higher expression of ITGA1 had an excellent accuracy in predicting 1-, 3-, and 5-year survival in LGG patients (Figure 3E,F). In addition, low expression of ITGA1 was associated with long progression-free interval and disease-specific survival in LGG patients (Figure 3G,H). The findings showed that ITGA1 was significantly correlated with an inferior prognosis in LGG patients.

### 3.3. ITGA1 Is Enriched in Immunomodulatory Pathways in LGG

**Figure 4:**
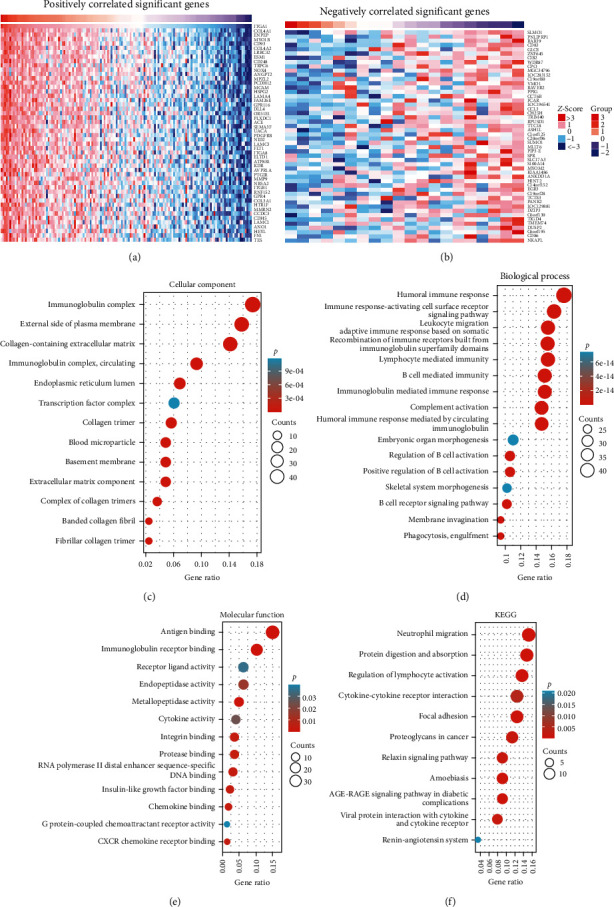
Functional enrichment of ITGA1-related genes in LGG. (A, B) Heat map of the top 50 genes that were positively and negatively associated with ITGA1. (C–E) GO enrichment analysis of cellular components, biological processes, and molecular functions that the ITGA1-associated gene involved. (F) KEGG enrichment analysis of signaling pathways that the ITGA1-associated gene involved.

To further reveal the biological function of ITGA1 in glioma progression, we screened for genes that were correlated with ITGA1 by differential expression analysis (Figure 4A,B). Enrichment analyses were performed for positively associated genes. Gene ontology (GO) enrichment analysis exhibited that they are mainly engaged in antigen binding, mediating immune regulation of B cells, immunoglobulin complex formation, and cytokine activation (Figure 4C–E). KEGG enrichment indicated that these genes were enriched in the pathway that regulates neutrophil migration, cytokine interactions, and lymphocyte activation (Figure 4F). These findings indicated that ITGA1 may be engaged in the immunomodulatory process in LGG.

### 3.4. ITGA1 Expression Correlates With Immunotherapy Response

**Figure 5:**
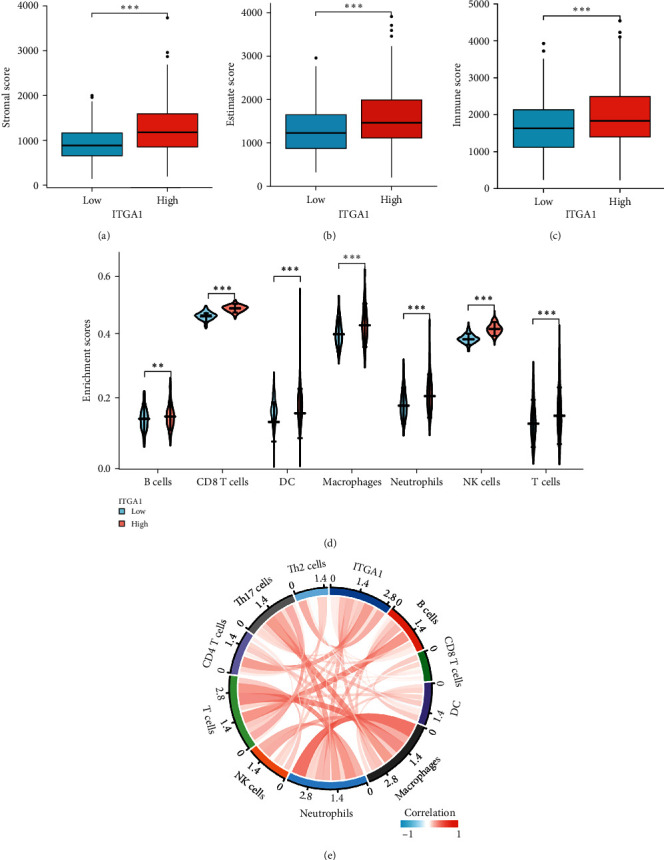
Immune score analysis of ITGA1 in LGG. (A–C) ITGA1 expression was positively related to estimate score in the TCGA LGG dataset. (D) Differential analysis of dendritic cells (DCs), neutrophils, CD8^+^ T cells, macrophages, natural killer cells, and B cells immune cells in relation to ITGA1 expression. (E) Correlation analysis between ITGA1 expression and immune cell infiltration (B cells, macrophages, neutrophils, DCs, Th2 cells, CD8^+^, CD4^+^, and NK cells). *⁣*^*∗∗*^*p*  < 0.01; *⁣*^*∗∗∗*^*p*  < 0.001.

**Figure 6:**
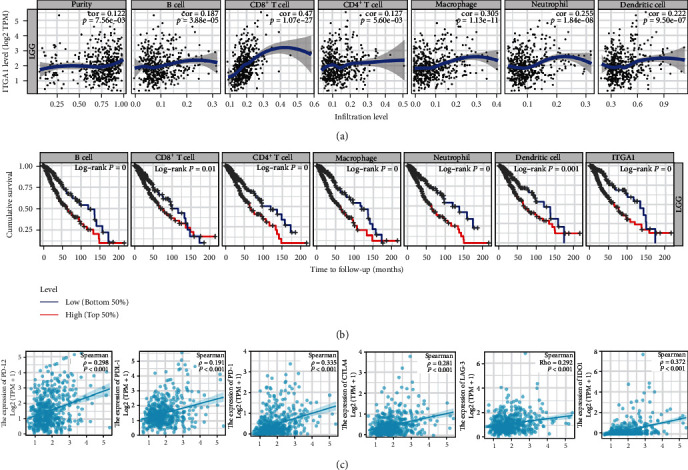
Correlation between ITGA1 expression and immune response. (A) High ITGA1 expression was associated with high infiltration levels of tumor purity (*r* = 0.122, *p*  < 0.0001), B cells (*r* = 0.187, *p*  < 0.0001), CD8^+^ T cells (*r* = 0.47, *p*  < 0.0001), CD4^+^ T cells (*r* = 0.127, *p*  < 0.0001), macrophages (*r* = 0.305, *p*  < 0.001), neutrophils (*r* = 0.255, *p*  < 0.001), and dendritic cells (*r* = 0.222, *p*  < 0.0001). (B) Cumulative survival analysis of ITGA1 expression, B cell, dendritic cell, CD4^+^ T cell, neutrophil, macrophage, and CD8^+^ T cell based on the TCGA LGG dataset. (C) ITGA1 expression was correlated with the level of PD-L2 (*ρ* = 0.298, *p*  < 0.001), with PD-L1 (*ρ* = 0.191, *p*  < 0.001), PD-1 (*ρ* = 0.335, *p*  < 0.001), CTLA4 (*ρ* = 0.281, *p*  < 0.001), LAG3 (*ρ* = 0.292, *p*  < 0.001), and IDO1 (*ρ* = 0.372, *p*  < 0.001).

To confirm the potential function of ITGA1 in tumor immunity, we performed the ESTIMATE methods to analyze the relation between ITGA1 and immunological infiltration based on TCGA dataset. It was shown that ITGA1 was positively correlated with an immune score, stromal score, and estimate score. (Figure 5A–C), indicating that ITGA1 plays a key role in LGG TME. We next employed the ESTIMATE algorithm to investigate the differences in the infiltrating immune cells in the TCGA dataset between ITGA1 high and low expression groups. These results showed that macrophages, neutrophils, B cells, monocytes, dendritic cells (DCs), and nature killer (NK) cells were significantly upregulated in the higher expressing ITGA1 group (Figure 5D), indicating that ITGA1 may contribute to the formation of an immunosuppressive microenvironment in LGG. Furthermore, we also analyzed the following relationships between immune cell infiltration and ITGA1 expression as follows by TIMER2.0. The expression of ITGA1 was positively related to the level of infiltration of CD4^+^ T cells, DCs, neutrophils, B cells, and NK cells (Figure 6A). Cumulative survival was analyzed to show that CD4^+^ T cells, DCs, neutrophils, B cells, and NK cells were significantly different in the low ITGA1 group. Immune cell infiltration was associated with a shorter prognostic viability time with increased ITGA1 expression, indicating further that highly infiltrated immune cells promote the formation of an immunosuppressive microenvironment and hence a poorer prognosis. (Figure 6B). We analyzed the correlation between ITGA1 expression with immune checkpoint-related genes. It was shown that the expression of ITGA1 was positive in correlation with PD-1, IDO1, PD-L2, LAG3, PD-L1, and CTLA4 immune checkpoints (Figure 6C). The findings further support the assumption that ITGA1 expression influences the TME immune response of LGG.

### 3.5. ITGA1 Promotes Glioma Cell Proliferation and Invasion

**Figure 7:**
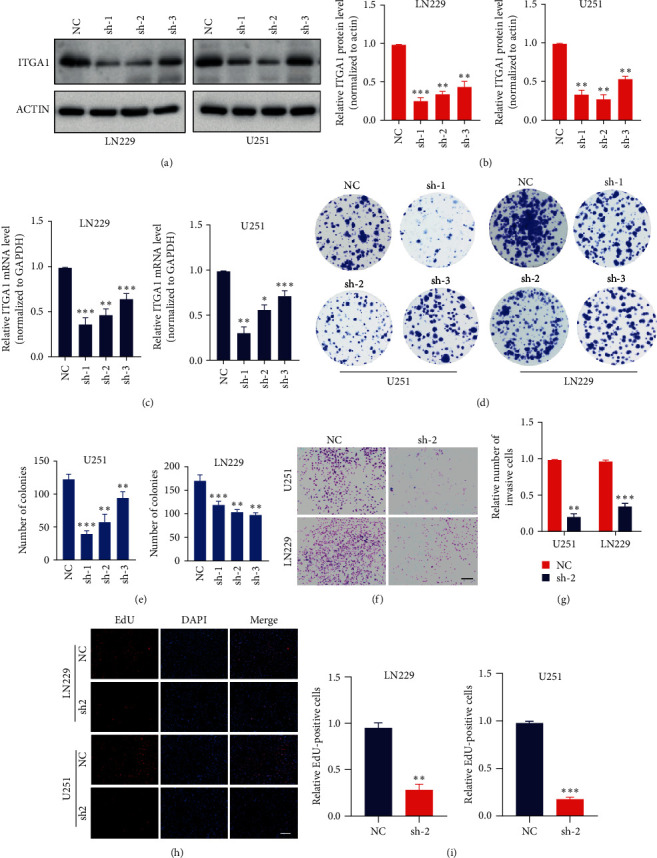
ITGA1 promotes the proliferation of glioma cells. (A) Glioma cell lines were constructed to stably downregulate ITGA1. (B) ITGA1 protein expression was statistically analyzed. (C) qPCR experiments were performed to analyze the expression level of ITGA1 mRNA. (D) Clone formation experiments were performed to detect the clone formation ability of glioma cells. (E) Clone formation was statistically analyzed by clone formation assay. (F) The cell invasion assay detects the ability of glioma cells to invade. Bar: 100 μm. (G) Cell invasion assay was statistically analyzed. (H) Detection of the proliferative capacity of glioma by EdU proliferation assay. Bar: 100 μm. (I) EdU proliferation was analyzed using statistic method. *⁣*^*∗∗*^*p*  < 0.01; *⁣*^*∗∗∗*^*p*  < 0.001.

In order to further investigate whether ITGA1 promotes the proliferation of tumor cells, we constructed glioma cell lines with stable downregulation of ITGA1 in LN229 and U251 cells, respectively. We first applied an immunoblotting assay to detect the expression level of ITGA1 (Figure 7A,B), and further verified the mRNA level by qPCR, indicating that the glioma cell lines with stably downregulated ITGA1 were successfully constructed (Figure 7C). In order to further verify whether the downregulation of ITGA1 would affect the clone formation ability of glioma cells, we performed clone formation assay, the results showed that it was significantly reduced after downregulation of ITGA1 (Figure 7D,E). Similarly, after known down of ITGA1, the invasion ability of glioma cells was inhibited (Figure 7F,G). EdU experimental results showed that known down of ITGA1 significantly inhibited the proliferation ability of U251 and LN229 cells (Figure 7H,I). Thus, high expression of ITGA1 in gliomas significantly promoted the proliferation of glioma cells.

## 4. Discussion

In this research, ITGA1 was found to be higher in LGG than in normal brain tissue in the level of expression. We also confirmed consistent results by qPCR and Western blot of glioma samples and cell lines. Therefore, ITGA1 expression was associated with an inferior prognosis in LGG patients. Functional enrichment showed that ITGA1 expression was linked to antigen binding, mediating immune regulation of B cells, and immunoglobulin complex formation, demonstrating the inhibitory role of ITGA1 in antitumor immunomodulation. Furthermore, we confirmed that ITGA1 expression is linked to the infiltration of immune checkpoint gene expression.

Integrins are dimeric heterodimers composed of membrane glycoproteins and noncovalently associated alpha and beta subunits that mediate intercellular signaling [19, 20]. ITGA1 is an important member of the integrin family and has previously been shown to play a role in tumorigenesis [21]. In this study, we analyzed ITGA1 on a pan-cancer basis and revealed that ITGA1 was highly expressed in many kinds of cancer, including metastatic melanoma, lung cancer, HCC, and gastric cancer. This is in consistent with the tumor-promoting role of ITGA1 in these cancers as reported before [13–15, 22]. We for the first time revealed that ITGA1 expression at mRNA levels was higher in GBM than in normal brain, which was also the case at ITGA1 protein levels as reflected by the Western blot and immunohistochemistry (IHC) analysis.

IDH1 mutation and 1p19q-Codel correlate with a good prognosis in glioma [23]. Further analysis showed that ITGA1 expression was high in LGG patients with IDH wildtype and 1p19q-Noncodel. Multifactor Cox regression analysis showed that high ITGA1 may be an independent risk factor for LGG patients. In addition, ROC analysis validated that high ITGA1 expression could be a sensitive predictor of 1-, 3-, and 5-year survival in LGG patients. These results suggest that ITGA1 is a good indicator for pathology diagnosis and prognostic assessment of LGG. Further investigations are required to verify the role of ITGA1 in glioma progression.

Enrichment analysis is a useful tool for predicting the function of genes [18]. Here we revealed that ITGA1 was involved in the biological processes, such as antigen binding, mediating immune regulation of B cells, neutrophil migration, and regulation of B cell activation signaling pathways, the majority of which are related to tumorigenesis and immune response [24]. Actually, previous studies have shown that other integrins like ITGA2, ITGA5, and ITGA11 play a significant role in immune regulation in the TME [25–27]. Thus, ITGA1 may contribute to the malignant progression of glioma by altering immune microenvironment.

ESTIMATE method is available online for analyzing the correlation between gene expression and the immune microenvironment [28]. In present study, a significantly positive relationship was revealed between ITGA1 expression and the estimate score, stromal score, and immune score in the TCGA LGG dataset. Moreover, we showed that high ITGA1 expression correlated with the infiltration of macrophage, neutrophils, CD8^+^ T cells, and NK. Macrophage contribute to tumor metastasis by producing immunosuppressive factors and promoting angiogenesis [29]. CD8^+^ T cells have the function of killing tumor cells and are the most important type of immune cells in LGG [30]. Our results show that in the ITGA1 high-expression group, there was a significant difference in CD8^+^ cell infiltration. Additionally, ITGA1 expression and immune cell high-infiltration were implicated with unfavorable prognosis in LGG. Therefore, the increased expression of ITGA1 in LGG may promote the formation of an immunosuppressive microenvironment, and thus, indicate a poor prognosis in LGG patients.

Immune checkpoint blockade is a promising strategy in tumor immunotherapy [31]. It has been shown that therapeutic inhibition of IDO1, CTLA-4, or PD-L1 significantly reduced the number of tumor-infiltrating T cells and significantly prolonged the survival in a mouse model of glioma [32]. Anti-PD-1/PD-L1 and anti-CTLA4 immunotherapy are promising treatment approach [32]. Here we evidence that ITGA1 expression was positively associated with the expression of PD-L1, CTLA4, PD-1, and PD-L2 and the infiltration of T cells, NK cells, and macrophages. Therefore, ITGA1 may be a potential target for immunotherapy in LGG patients.

## 5. Conclusions

The expression of high ITGA1 is associated with an inferior prognosis in LGG. ITGA1 expression was altered the glioma immune environment and promoted the expression of immune checkpoint-related genes and immune cell infiltration. In vivo experiments showed that overexpression of ITGA1 promoted glioma cell invasion. All results indicated that ITGA1 is a target for potential immunotherapy of LGG.

## Data Availability

All data supporting the conclusions of this study are included in the article.

## Supporting Information

Supporting Information 1 Figure S1: Immunohistochemistry statistical was analyzed in nontumor brain tissues and WHO grade 2 glioma tissues.

Supporting Information 2 Table S1: Glioma tissue sample information, Table S2: Univariate and Multivariate Cox regression analyses based on the CGGA dataset.
