# Supplementary material for: ITGA1 Promotes Glioma Cell Proliferation and Affects Immune Cell Infiltration in Low-Grade Glioma
Source: Mediators Inflamm. 2024 Oct 29;2024:6147483. doi: 10.1155/2024/6147483 (PMC11537738; doi:10.1155/2024/6147483)

**Supplementary Figure 1**

Immunohistochemistry statistical was analyzed in nontumor brain tissues and WHO grade 2 glioma tissues. (** p < 0.01)


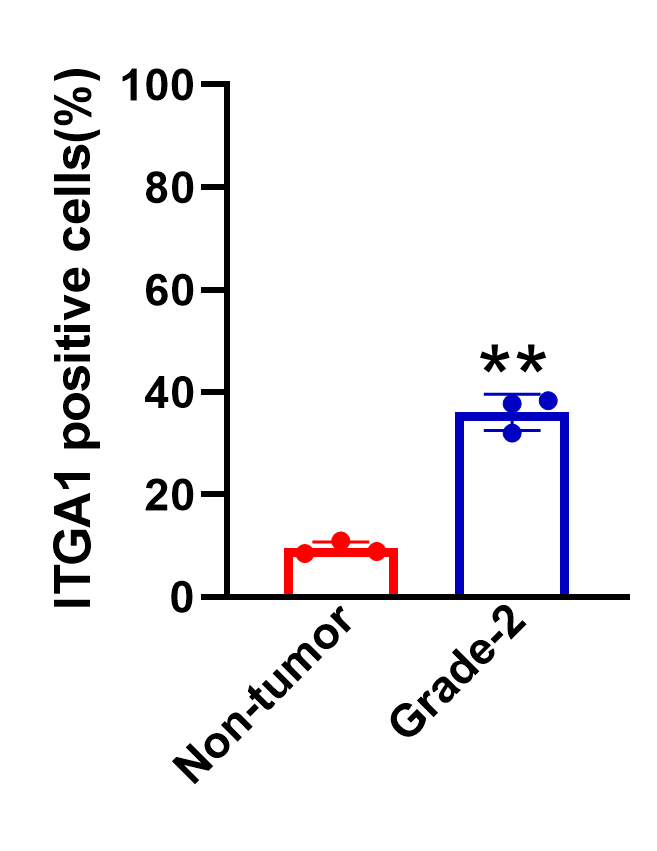

Supplement: Supporting Information 1 — Figure S1: Immunohistochemistry statistical was analyzed in nontumor brain tissues and WHO grade 2 glioma tissues. [file 6147483.f1.docx]
