# Supplementary material for: ITGA1 Promotes Glioma Cell Proliferation and Affects Immune Cell Infiltration in Low-Grade Glioma
Source: Mediators Inflamm. 2024 Oct 29;2024:6147483. doi: 10.1155/2024/6147483 (PMC11537738; doi:10.1155/2024/6147483)
Supplement: Supporting Information 2 — Table S1: Glioma tissue sample information, Table S2: Univariate and Multivariate Cox regression analyses based on the CGGA dataset. [file 6147483.f2.docx]

**Table S1 Glioma tissue sample information**

| Case No | Code No | Gender | Age(years) | Used for | Grade |
| --- | --- | --- | --- | --- | --- |
| 1 | 1864096 | M | 47 | qPCR, WB | Non-tumor |
| 2 | 1780327 | F | 55 | qPCR,WB | Non-tumor |
| 3 | 3156038 | F | 36 | WB | Non-tumor |
| 4 | 1742499 | M | 26 | qPCR,WB | Non-tumor |
| 5 | 1250590 | M | 55 | WB | Non-tumor |
| 6 | 1330482 | M | 75 | qPCR,WB | Non-tumor |
| 7 | 1668083 | F | 44 | qPCR | Non-tumor |
| 8 | 1685462 | F | 72 | qPCR | Non-tumor |
| 9 | 1752439 | F | 46 | qPCR,WB | Grade 2 |
| 10 | 2206546 | F | 37 | WB | Grade 2 |
| 11 | 1701564 | F | 62 | qPCR,WB | Grade 2 |
| 12 | 1844045 | F | 48 | qPCR,WB | Grade 2 |
| 13 | 1799226 | M | 43 | qPCR,WB | Grade 2 |
| 14 | 1786094 | M | 31 | qPCR | Grade 2 |
| 15 | 1771635 | F | 43 | qPCR | Grade 2 |
| 16 | 3128869 | F | 67 | WB | Grade 2 |
| 17 | 1698761 | M | 54 | WB | Grade 2 |
| 18 | 2089401 | M | 51 | WB | Grade 3 |
| 19 | 1626742 | M | 64 | WB | Grade 3 |
| 20 | 1797389 | F | 55 | WB | Grade 3 |
| 21 | 1830046 | F | 49 | qPCR,WB | Grade 3 |
| 22 | 201282173 | M | 69 | WB | Grade 3 |
| 23 | 3026457 | M | 54 | WB | Grade 3 |
| 24 | 1686765 | M | 53 | qPCR,WB | Grade 3 |
| 25 | 1615001 | M | 71 | qPCR | Grade 3 |
| 26 | 1412500 | F | 56 | qPCR | Grade 3 |
| 27 | 1413682 | F | 56 | qPCR | Grade 3 |
| 28 | 1842250 | M | 59 | qPCR | Grade 3 |
| 29 | 2048241 | F | 52 | WB | Grade 4 |
| 30 | 1875264 | F | 67 | qPCR,WB | Grade 4 |
| 31 | 3088419 | M | 69 | WB | Grade 4 |
| 32 | 1743383 | M | 53 | qPCR,WB | Grade 4 |
| 33 | 1692750 | M | 57 | qPCR,WB | Grade 4 |
| 34 | 1672752 | M | 55 | qPCR,WB | Grade 4 |
| 35 | 1741779 | F | 34 | qPCR | Grade 4 |
| 36 | 1746637 | M | 68 | qPCR | Grade 4 |
| 37 | 1771079 | M | 41 | qPCR | Grade 4 |

**Table S2 Univariate and Multivariate Cox regression analyses based on the CGGA dataset**

| Characteristic | HR | P-Value | |  | | HR | | 95%CI | | P-Value | |
| --- | --- | --- | --- | --- | --- | --- | --- | --- | --- | --- | --- |
| Univariate |  |  | Multivariate | |  | |  | |  | |  |
| Age | 1.72 | 0.034 | Age | | 1.19 | | 0.69-2.04 | | 0.534 | |  |
| Chemo-status | 0.43 | 0.001 | Chemo-status | | 0.45 | | 0.26-0.76 | | 0.003 | |  |
| IDH-mutation | 3.54 | 0.000 | IDH-mutation | | 1.69 | | 0.96-2.96 | | 0.067 | |  |
| 1p19q-codel | 6.39 | 0.000 | 1p19q-codel | | 5.39 | | 2.52-11.5 | | 0.000 | |  |
| ITGA1 | 1.76 | 0.026 | ITGA1 | | 1.85 | | 1.10-3.12 | | 0.021 | |  |
